# Acute spontaneous non-hemorrhagic adrenal infarction with systemic lupus erythematosus and antiphospholipid antibody syndrome: A case report

**DOI:** 10.1097/MD.0000000000039092

**Published:** 2024-08-02

**Authors:** Chunxiao Liang, Taichun Qiu, Zhongyan Lu, Bing Ming, Dongmei Xie, Fei Wang, Qing Zou

**Affiliations:** a Department of Radiology, People’s Hospital of Deyang City, Sichuan, China; b Department of Interventional Radiology, People’s Hospital of Deyang City, Sichuan, China.

**Keywords:** adrenal infarction, antiphospholipid antibody syndrome, computed tomography, magnetic resonance imaging, systemic lupus erythematosus

## Abstract

**Rationale:**

Adrenal infarction (AI) is a rare type of adrenal damage, which is relatively common in systemic lupus erythematosus, antiphospholipid antibody syndrome (APS) and pregnancy. The diagnosis of AI is mainly by computed tomography (CT) and magnetic resonance imaging, but is easily confused with other adrenal disease. Hence, this report details a condition of AI with systemic lupus erythematosus, APS and made a differential diagnosis from imaging.

**Patient concerns:**

We report a case of a 55-year-old woman with pain in her fossa axillaries and inguinal regions. Then CT scan disclosed bilateral adrenal diseases, and the patient was diagnosed with systemic lupus erythematosus, APS and AI after additional autoimmune examinations.

**Diagnoses:**

The patient was diagnosed as systemic lupus erythematosus with lupus nephritis, hematological damage and oromeningitis, APS, AI and secondary blood coagulation disorders.

**Interventions:**

The patient was treated with methylprednisolone, hydroxychloroquine and low molecular heparin.

**Outcomes:**

The patient relieves and remains well 1 year after treatment.

**Lessons subsections:**

AI can be divided hemorrhagic and non-hemorrhagic, with bilateral lesions more common. In our case, the AI was bilateral, partially involved and non-hemorrhagic, and the “cutoff sign” was first put forward in CT, which might assist the diagnosis.

## 1. Introduction

Adrenal gland is a retroperitoneal organ, with special anatomical features. It has 3 feeding arteries and only one draining vein, a “vascular dam” structure,^[1]^ which is an essential inducement of acute adrenal disease.

Trauma, neoplasm, infection, hemorrhage, and infarction^[2]^ are the causes of acute adrenal diseases. These conditions can lead to acute adrenal failure, a potentially fatal condition. Acute adrenal infarction (AI) is very rare and easily overlooked, especially in patients without adrenal insufficiency, which is diagnosed mainly by imaging. AI was relatively common in antiphospholipid antibody syndrome (APS),^[3–6]^ pregnancy,^[7]^ and also existed in Crohn disease,^[8]^ COVID-19,^[9]^ myelodysplastic/myeloproliferative neoplasm,^[10]^ substance use (e.g., methamphetamine),^[11]^ heparininduced thrombocytopenia^[12]^ and aortic dissection,^[2]^ etc. However a large portion of individuals originally manifested signs of AI before the primary disease was identified.^[4,10,13]^ Therefore, it is crucial for the diagnosis of AI in imaging. The purpose of this paper is to describe a case of AI, provide an overview of the imaging features associated with autoimmune disorders, and then carry out a differential diagnostic study.

## 2. Case report

**Figure 1.**
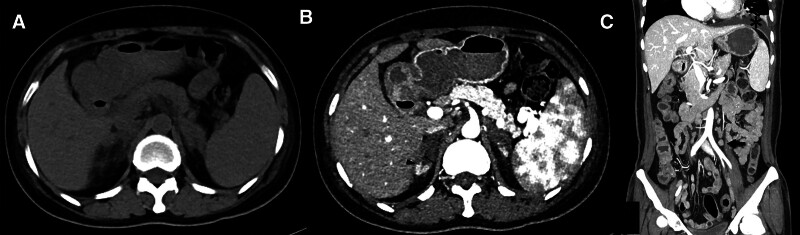
CT features of non-hemorrhagic adrenal infarction. (A) Non-contrast CT scan reveals the thickening adrenal gland with surrounding stranding. (B) Enhanced CT shows the poor enhancement of lesion, “cutoff sign” (arrowhead) and “capsular sign” (arrow). (C) Coronal enhanced CT image demonstrates the enlargement of para-aortic lymph nodes (arrow) and pleural effusion (asterisk). CT = computed tomography.

The patient information was anonymized and de-identified before analysis. Informed consent for publication of the case was obtained. A 55-year-old female farmer had pain in her fossa axillaries and inguinal regions for 3 days, aggravated and accompanied with low-grade fever for 1 day. She was also fatigue, nausea and walked unsteadily. Ten days ago, she underwent cholecystectomy at our hospital for calculous cholecystitis. Denied any chronic underlying conditions. Lymphadenopathy of armpit and groin was discovered in physical examination, which was confirmed in follow-up ultrasound with partial cortical thickening. Then the blood routine examination disclosed the neutrophilic granulocyte percentage was 90.0%, and high-sensitivity C-reactive protein was 173.84 mg/L. In the coagulation examination, the international normalized ratio was 1.35. And the prothrombin time, activated partial thromboplastin time, fibrinogen degradation products and d-dimer level were abnormal, their values were 15.4s, 55.4s, 7.16 mg/L and 2.46 mg/L, respectively. Additionally, blood amylase and lipase levels increased to 212 U/L and 155 U/L, respectively. Then the abdominal enhanced computed tomography (CT) was performed, but the images revealed a normal pancreas and aberrant adrenal glands, characterized by thickening and poor enhancement, with peripheral stranding. The adrenal glands were partially involved, with the distal of medial and lateral limbs survival, and there was a clear boundary between the sicked and normal area of adrenal gland, named “cutoff sign” (Fig. 1). The spleen and abdominal lymph nodes were enlarged, accompanied by small pleural effusion. After hematology consultation, additional laboratory examination showed that the anticardiolipin-IgG, anti-β2 glycoprotein antibody-IgA and other autoimmune antibodies were significantly abnormal (Table 1). 24-hour urine protein was 0.85g/24h. Fungal serological tests, cytomegalovirus DNA and Epstein-Barr virus DNA, adrenocorticotrophic hormone, sex hormone and cortical test were normal. After 3 months, anticardiolipin-IgG, anti-β2 glycoprotein antibody-IgA were also abnormal. Then the patient was diagnosed with systemic lupus erythematosus with lupus nephritis, hematological damage and oromeningitis, APS, AI and secondary blood coagulation disorders.

**Table 1:** Laboratory data.

Variables	Results
aCL (IgG) (IU/mL)	49.71 (<12)
Anti-β2 GPI (IU/mL)	33.73 (<24)
Immunoglobulin A (g/L)	2.55 (0.7–4)
Immunoglobulin G (g/L)	13.40 (7–16)
Immunoglobulin M (g/L)	0.79 (0.4–2.3)
C3 complement (g/L)	0.43 (0.8–2)
C4 complement (g/L)	0.07 (0.1–0.4)
ANA	Positive
ANA titer	1: 3200
ANA karyotype	Homogeneous
Anti-dsDNA antibody	Positive
Anti-smith antibody	Positive
Anti-SS-A antibody	Positive
Anti-SS-B antibody	Positive
Anti-cenp-b antibody	Positive

aCL = anticardiolipin antibody, ANA = antinuclear antibodies, anti-β2GPI = anti-beta2 glycoprotein I antibody, dsDNA = anti-double-stranded DNA antibody.

## 3. Results

**Figure 2.**
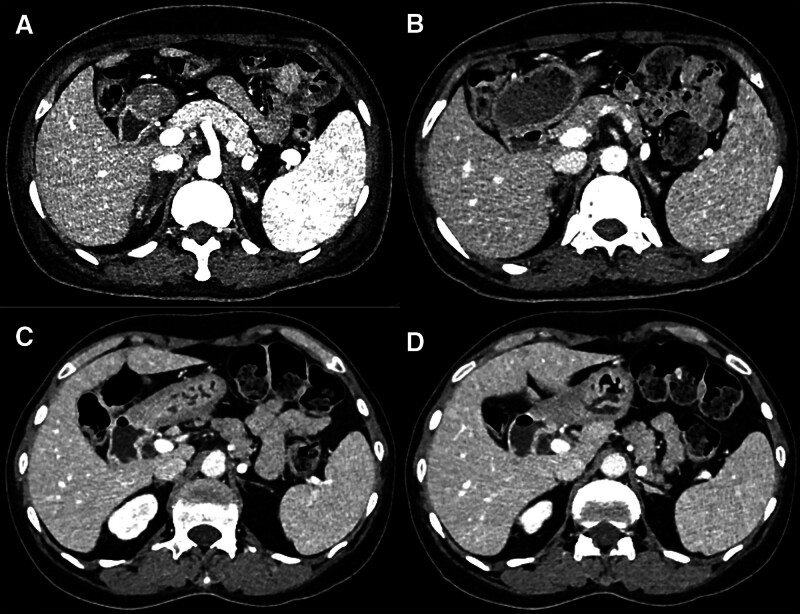
The changes of adrenal infarction in the subsequent CT reviews. (A and B) The first and second CT reviews show the adrenal glands atrophy gradually. (C and D) The last CT review after 1 yr shows the obviously absorbed of lesion, with a scanty amount of adrenal gland remnant. CT = computed tomography.

The patient was treated with methylprednisolone, hydroxychloroquine and low molecular heparin during hospitalization. Subsequently, she readmitted in our hospital for cyclophosphamide chemotherapy, 7 in total. During treatment, she reviewed abdominal CT scans for thrice (Fig. 2). The first review was performed 10 days after treatment initiation, revealing slight adrenal atrophy and clearer surrounding fat space. The second review, conducted 1 month later, showed some lesion contraction with absorption of peripheral exudation after 2 chemotherapies. The last scan performed 1 year later with chemotherapy ended, the area of adrenal disease was obviously atrophy, and the remnant part was normal. Moreover, the pleural effusion, enlarged spleen and lymph nodes disappeared. These findings manifested the treatment was effective, and the patient symptoms had been in remission.

## 4. Discussion

AI had been reported in autoimmune diseases, mainly included systemic lupus erythematosus and APS. The Pathogenesis of AI is possibly due to microvascular or single-draining vein thrombosis.^[8]^ APS is a condition characterized with recurrent thrombotic events, recurrent abortion and antiphospholipid antibodies.^[14,15]^ The late endosomal and lysosomal lysobisphosphatidic acid in the adrenal cortex cells with rich cholesterol are suffered attack by antiphospholipid antibodies, which promotes a procoagulant condition. Meanwhile, antiphospholipid antibodies can damage the endotheliocytes and prompt the shifting of Annexin V by up-regulation of E-selectin and vascular cell adhesion molecule-1, generating microvascular thrombosis.^[4]^ Owing to the “vascular dam” structure, internal adrenal pressure increased secondary to microthrombosis, that can easily lead to subsequent hemorrhage.

Studies showed that patients with AI often presented as abdominal pain, fever, vomiting, diarrhea, and weakness.^[12,16]^ Unlike previous cases, the patient in our case experienced pain in fossa axillaries and inguinal regions, possibly indicative of radiating pain. The high serum amylase level can present in renal failure, salivary gland disorders and so on,^[17]^ not only acute pancreatitis. In our case, the patient was diagnosed with lupus nephritis, this may be the reason for hyperamylasemia.

Imaging plays a crucial part in the diagnosis of AI of autoimmune diseases, and it can divide into hemorrhagic and non-hemorrhagic infarction.^[2]^ Whether hemorrhagic or non-hemorrhagic AI, it could be unilateral or bilateral, partial or total involvement, but bilateral is more common.^[5]^ Hemorrhagic AI had a higher morbidity. On non-contrast CT and magnetic resonance imaging (T1 weighted image), infarcted adrenal glands with bleeding were enlarged and iso- or slightly hyperintensity, and the surrounding fat got involved. However, in the enhanced examination, it difficult to assess whether the diseased area had any enhancement for the mask of bleeding signal. The non-hemorrhagic AI had similar changes in the morphology, and the density was decreased slightly in non-contrast CT, and slightly hyperintense in T2 weighted image (T2WI) secondary to edema. In addition, the lesion was also high-intensity on diffusion-weighted image due to restricted diffusion water molecules.^[6]^

On contrast CT/magnetic resonance imaging, the non-hemorrhagic AI had obviously poor or no enhancement.^[6]^ Moschetta et al had found the “capsular sign,” with 100% specificity, in diagnosis of acute adrenal ischemia.^[18]^ And the “capsular sign” is likely resulting from the residual blood supply of the adrenal capsular veins.^[18]^

In our report, the AI is non-hemorrhagic, with only CT examination. Bilateral adrenal glands were partially involved, with “cutoff sign” and “capsular sign,” which were also observed in previous reports.^[8]^ The distal of adrenal medial and lateral limbs had normal perfusion, forming a “cutoff sign.” We hypothesized that might be related to less involvement of distal microvascular plexus or collateral vessel formation. Consistent with previous studies,^[13,19]^ the adrenal gland atrophy was detected in the follow-up CT. When 90% of adrenal gland tissue is destroyed, the symptoms and indicators of adrenal insufficiency would be obvious.^[5]^ Because the adrenal glands were partially involved, lower than 90%, there were no classic symptoms of adrenal insufficiency in our case. Therefore, the diagnosis of AI based on clinical symptoms and laboratory examinations is challenging. Nevertheless, for the highly suspicious AI in abdominal CT, the diagnosis was well-established. The patient received timely treatment, and her condition was controlled. In the follow-up CT, the residual adrenal gland showed normal perfusion.

However, the manifestation of AI is arduous to distinguish from other acute adrenal diseases. Adrenal hematoma and intratumoral adrenal hemorrhage are often unilateral and round or oval shaped,^[20]^ which is a discrepancy with hemorrhagic AI. And adrenal hematomas often have history of trauma or stress.^[2]^ Meanwhile, tumoral density or signal is heterogeneous, and the enhancement of neoplastic tissue also exists, especially in the subtraction image.^[2]^ For non-hemorrhagic AI, whose imaging features are similar to adrenal infection in some extent. And the majority of adrenal infection is adrenal tuberculosis.^[21]^ Adrenal tuberculosis similarly tends to affect both adrenal glands,^[2]^ but differs from AI in its uneven thickening, often presenting with a rugged surface or nodular appearance.^[22]^ Additionally, adrenal tuberculosis exhibits heterogeneous density on non-contrast CT, with caseous necrosis and calcification.^[22,23]^ Furthermore, reduced, uneven and ring enhancement often occurs.^[23]^ The majority of patients may have tuberculosis of other organs in CT, which can also contribute to the diagnosis of adrenal tuberculosis.^[22]^

## 5. Study limitations

For the retroperitoneal anatomical location of adrenal glands and the potential aggravation of adrenal damage, the histopathology examination is difficult to accomplished. Nonetheless, the typical manifestation in CT and the pivotal laboratory results assisted in the correct and decided diagnosis. In addition, the etiology of AI is diverse, whether there are some differences in the image characteristics is unknown. So, future studies are needed to answer the doubt.

## 6. Conclusion

AI is rare, commonly seen in patients with autoimmune diseases, especially systemic lupus erythematosus and APS. The “cutoff sign” of our article is first raised, indicating the diagnosis of partially involved non-hemorrhagic AI with autoimmune diseases.

## Author contributions

**Conceptualization:** Chunxiao Liang, Taichun Qiu, Fei Wang

**Investigation:** Chunxiao Liang, Zhongyan Lu, Bing Ming, Dongmei Xie

**Writing – original draft:** Chunxiao Liang, Taichun Qiu

**Writing – review & editing:** Chunxiao Liang, Qing Zou, Taichun Qiu
